# Effects of small-sided games training programs on physiological and physical adaptations of youth basketball players: A systematic review

**DOI:** 10.1177/00368504241231657

**Published:** 2024-03-06

**Authors:** Tingyu Li, Qi Xu, Hugo Sarmento, YongXing Zhao, Rui Miguel Silva, Filipe Manuel Clemente

**Affiliations:** 1 Sport physical activity and health research & innovation center, Viana do Castelo, Portugal; 2 Department of Biomechanics and Sport Engineering, Gdansk University of Physical Education and Sport, Gdańsk, Poland; 3 Faculty of Sport Sciences and Physical Education, University of Coimbra, Research Unit for Sport and Physical Activity (CIDAF), Coimbra, Portugal; 4 College of Physical Education, Chizhou University, Chizhou, Anhui, China; 5 Escola Superior Desporto e Lazer, 112031 Instituto Politécnico de Viana do Castelo, Rua Escola Industrial e Comercial de Nun’Álvares, Viana do Castelo, Portugal

**Keywords:** Basketball, athletic performance, physical fitness, sports training, drill-based games, systematic review

## Abstract

The primary objective of this study was to systematically investigate the physiological and physical fitness adaptations resulting from small-sided games (SSGs) training programs in basketball players competing at youth competitive levels, as compared to other training approaches and/or control groups. To achieve this, we conducted a literature search on PubMed, Scopus, SPORTDiscus, and Web of Science, adhering to the Preferred Reporting Items for Systematic Reviews and Meta-Analyses guidelines. From the initial 626 studies retrieved, five were considered eligible for the current study. Among the five included articles, four conducted comparisons between the effects of SSGs and running-based high-intensity interval training. Regarding this, the four studies revealed a significant improvement in the final velocity during the 30–15 Intermittent Fitness Test, ranging from 4.07% to 7.29% following SSG-based interventions. This improvement was not significantly different from the comparator group. Additionally, two studies indicated that the SSGs group showed a significant advantage in change-of-direction time, with improvements ranging from −2.11% to 6.69% after interventions, and these results were not significantly different from the comparator group. However, the effects on repeated sprint ability yielded contradictory findings; two studies reported significant improvements ranging from −5.00% to −2.16%, while two others did not show significant effects following SSGs-based interventions. Similarly, in the linear sprint, the results of SSGs-based interventions were inconsistent. In summary, based on the available research, it can be concluded that SSG-based training is effective in significantly enhancing aerobic performance and change of direction, comparable to alternative approaches. However, the effects on repeated sprint ability and sprint performance are not consistently demonstrated.

## Introduction

Basketball is a demanding sport that requires a combination of physical and physiological abilities.^1–3^ From a physiological standpoint, the cardiovascular system plays a crucial role in supporting players’ endurance, which is vital to keep up with the fast-paced nature of the game.^4^ In this regard, basketball relies heavily on both anaerobic and aerobic energy metabolism to sustain high-performance levels during repeated instances of high-intensity actions throughout match-play.^5,6^ The intermittent nature of basketball, with alternating bouts of high-intensity activity and periods of low- to moderate-intensity activity, further emphasizes the importance of anaerobic fitness.^7^ This requirement stems from the necessity of executing explosive movements like jumping and sprinting during the game.^8,9^

Considering the above-mentioned demands, high-intensity interval training (HIIT) involving both metabolisms (aerobic and anaerobic) has established itself as a common training method for enhancing aerobic fitness, anaerobic power, and the ability to repeatedly make intense efforts, coping with the scenario of the game.^10,11^ Characterized by intense and intermittent exercises interspersed with recovery periods, HIIT offers time efficiency while ensuring a well-established efficacy in the physical performance of basketball players.^12,13^

HIIT can be used in different ways, considering the training regimen and the manipulation of variables such as periods of work, recovery, intensity, or volume.^10^ Moreover, HIIT can be employed in different modes, going beyond the common running-based approach.^14^ In fact, one of the alternatives is using game-based scenarios, with popular small-sided games (SSGs) being one such option.^15^ These games represent an intense yet enjoyable training method that allows for providing multiple stimuli, including technical/tactical aspects, as well as physiological and physical elements.^16^

SSGs maintain the dynamics of the official format of play while using task constraints such as format (e.g. numerical relationships), court configuration (e.g. court size and width:length ratio), intrapersonal and interpersonal coordination (e.g. limiting the number of actions), or timing constraints (e.g. limiting the time available to perform specific tasks) to adjust the demands of the games to the operative objectives set by the coach for the session.^17^

Due to the fact of regularly being characterized by smaller formats (e.g. 1v1, 2v2) performed in smaller pitches (e.g. half of the court), these games impose, as a consequence, an adjustment in behavior that ultimately intensifies the physical demands (e.g. distances run at high velocity, high-intensity accelerations, and decelerations), resulting in an intensified physiological response (e.g. intense heart rate responses, typically above 85% of maximal heart rate, greater blood lactate concentrations).^18^ While considering the nature of adaptations as acute (immediate during the exercise), the recurrence of these drills over training programs and time can also impact chronic physical adaptations in response to the accumulated acute loads.^19^ As observed in other HIIT forms (e.g. running), physical adaptations are also being observed in those exposed to SSG training programs.^20^

The growing interest in using SSGs as an alternative or complementary approach to running-based HIIT has increased the number of experimental parallel and controlled studies comparing the physical performance adaptations in response to SSGs versus other training approaches.^12,20,21^ Although markedly more variable than running-based results due to the contextual dynamic of the game, which implies greater within-player variability with respect to key acute load outcomes,^22^ it has been interestingly observed that SSGs and running-based HIIT have similar effects on physical performance adaptations, such as aerobic performance or anaerobic power.^12,20,21^

Despite the growing evidence and two reviews dedicated to acute physiological and physical responses during basketball SSGs,^6,18^ there is no dedicated systematic review comparing SSGs and other training methods with respect to chronic physical performance adaptations in basketball players under the age of 18. In fact, during the developmental stage of young and youth basketball players, the use of SSG forms can be particularly interesting for both stimulating technical abilities and consolidating tactical behaviors,^23^ while providing a necessary physical and physiological stimulus that prepares the players for the demands of the game.^24^ Due to the specificity of young and youth populations, establishing the criteria under 18 is an attempt to focus data extraction and analysis on those in nonprofessional or adult competitive scenarios.

Given the former, this systematic study aims to review and offer insights into the relative effectiveness of different training approaches for youth basketball players, enabling coaches and trainers to make informed decisions tailored to the specific needs and objectives of their athletes. Furthermore, this systematic review aims to identify any existing gaps in the current literature, shedding light on areas that require further investigation and research.

## Methods

### Protocol registration

This systematic review adhered to the guidelines set forth by Preferred Reporting Items for Systematic Reviews and Meta-Analyses (PRISMA) 2020.^25^ The protocol for this review was registered on the Open Science Framework (OSF) with the code number DOI 10.17605/OSF.IO/YN78J on 24 June 2023. Interested parties can access the protocol via the designated web address: https://osf.io/yn78j/. The review also adhered to the recommended guidelines for reporting systematic reviews in sports sciences.^26^ Our a priori protocol underwent no post hoc alterations. It is important to note that our protocol was not published in a peer-reviewed journal.

### Eligibility criteria

In conducting this systematic review, we included original contributions accessible through peer-reviewed journals or those currently in the prepublication phase. Our scrutiny maintained a broad perspective by refraining from imposing language restrictions on the considered articles. The criteria for eligibility, structured according to the PICOS framework, are detailed in the ensuing Table 1. For non-English articles, we have invited native speakers proficient in the original language of the article to assist in translating both the abstract and, if necessary, the full text.

**Table 1.** Eligibility criteria.

	Inclusion criteria	Exclusion criteria
Population	This systematic review included basketball players in the youth categories, typically under the age of 19. No restrictions were imposed based on gender, training status, or competitive level.	This systematic review focused exclusively on adult basketball players. Players who were injured or ill, as well as participants involved in sports other than basketball, were not considered in the review.
Intervention	Players exposed to structured small-sided game (SSG) training programs without any restrictions on the duration or frequency of the training program. Additionally, there were no limitations on the training volume or intensity.	Participants who underwent a combination of SSGs and other training interventions.
Comparator	The review incorporated two types of control groups: passive control groups, which were not exposed to any other training interventions and maintained their regular training habits in the field, and active control groups, which were exposed to alternative exercise programs not involving SSGs training.	Groups that included an alternative method in conjunction with SSGs (e.g. running-based HIIT + SSGs) were excluded from the analysis.
Outcomes	The systematic review evaluated diverse chronic physical fitness outcomes, measured both before and after intervention, representing a comprehensive physical adaptation. These outcomes included endurance performance, speed, change-of-direction measures, muscular strength, power measures, and balance. Additionally, the review examined body characteristics or composition, including body mass index, fat mass, and lean mass. Moreover, health-related markers, such as biochemical markers and inflammatory markers, were also considered in the evaluation.	The systematic review explicitly excluded acute physiological and/or physical responses (e.g. immediate responses to exercise or single sessions). Furthermore, the review did not encompass socio/psychological factors or technical/tactical considerations in its evaluation.
Study design	The systematic review encompassed both two-arm study designs and multiarm study designs.	The systematic review excluded studies with no control group or parallel group.

HIIT: high-intensity interval training.

### Information sources

For the exploration phase, our systematic review engaged various databases, specifically: (i) PubMed, (ii) Scopus, (iii) SPORTDiscus (EBSCOHost), and (iv) Web of Science. The search endeavors were conducted on May 12, 2023, by HS and FMC. Complementing the database exploration, manual searches were undertaken on the cited references of the encompassed studies to pinpoint titles with potential relevance.

Subsequently, the abstracts of the identified articles underwent scrutiny to ascertain their alignment with preestablished inclusion criteria. Should the need arise, a more in-depth examination of the complete texts was carried out to gauge their suitability for inclusion in the systematic review.

### Search strategy

The formulation of the search strategy involved the automated utilization of Boolean operators AND/OR. In an effort to optimize the identification of pertinent studies, no filters or constraints were imposed on the publication date or language. The search strategy was implemented through the incorporation of the following codes:

[Title/Abstract] “basket*” AND [All fields/Full text] “small-sided games*” OR SSG* OR drill-based OR “constrained games” OR sided-game*.

Table 2 provides a comprehensive overview of the entire search strategy employed in this review.

**Table 2.** Full search strategy for each database.

Database	Specificities of the databases	Search strategy	Number of articles
PubMed	Search for title and abstract also includes keywords	(basket*[Title/Abstract]) AND (“small-sided games*” OR SSG* OR drill-based OR “constrained games” OR “sided-game*”)	46
Scopus	Search for title and abstract also includes keywords	(TITLE-ABS-KEY (basket*) AND ALL (“small-sided games*” OR ssg* OR drill-based OR “constrained games” OR sided-game*))	352
SPORTDiscus	Search for title and abstract also includes keywords	TI basket* AND TX (“small-sided games*” OR SSG* OR drill-based OR “constrained games” OR sided-game*)	157
Web of Science	Search for title and abstract also includes keywords and its designated “All Fields”	basket* (Title) and “small-sided games*” OR SSG* OR drill-based OR “constrained games” OR sided-game* (All Fields)	91

### Selection process

The screening process for the retrieved records, encompassing titles and abstracts, was independently conducted by two authors (HS and FMC). Furthermore, they individually evaluated the full texts of the chosen records. In instances of discord, a collaborative reassessment was initiated to achieve consensus. If unanimity proved elusive, a third author (TL) was consulted for the ultimate decision.

The arrangement of record management, inclusive of duplicate removal, was executed through EndNote X9.3.3 software (Clarivate Analytics, Philadelphia, PA, USA). To ensure a thorough record management, a combination of automated and manual methods was employed.

### Data extraction process

Authors TL and QX autonomously executed the data collection process. In the event of discrepancies or disagreements, FMC assumed the role of arbitrator to facilitate resolution. To enhance the efficiency of this procedure, a specialized Microsoft^®^ Excel datasheet was formulated, encapsulating all relevant data and key information gleaned from the chosen studies.

### Data items

The systematic review's data collection procedure spanned a diverse array of participant-related and contextual information. Essential particulars, including the publication date, primary research objective, sample size, country of origin, age, gender, clinical details (if relevant), and competitive level, were gathered. This compilation of a comprehensive participant-related dataset ensured a comprehension of the study populations under scrutiny.

Moreover, detailed intervention-related information was compiled to elucidate the nuances of the SSGs’ training programs. Specifics encompassed the timing of the competitive season, program duration, training frequency, adherence to the regimen, and various training regimen parameters, such as duration, repetitions, rest, intensity, frequency, and density. Additionally, factors such as the rules of play, format of play, and pitch size were all considered, contributing to a comprehensive understanding of the training interventions.

Comparators held a pivotal significance in this systematic review, and their details were recorded. Both passive control groups, maintaining their regular training habits without receiving additional interventions, and active control groups, subjected to alternative exercise programs unrelated to SSG training (e.g. running-based HIIT), were taken into account. Particulars regarding the nature of the exercise, intensity, and volume of the active control groups were documented, facilitating a comprehensive comparison among diverse training approaches.

This review assessed a diverse range of outcomes spanning physical fitness and health-related parameters. Physical fitness outcomes encompassed different variables, including endurance-related measures such as maximal oxygen uptake and maximal aerobic speed, neuromuscular-related indicators like muscular power and strength, and speed and change-of-direction-related measures such as sprint performance and change-of-direction performance. Furthermore, the evaluation included balance and mobility-related measures, encompassing both dynamic and static aspects of balance. The primary outcomes extracted for analysis included baseline and postintervention timing points.

The health-related outcomes delved into various aspects, encompassing body characteristics and composition, which involved measurements like body mass index, lean mass, and fat mass. Additional examinations covered blood pressure, echocardiographic assessments such as cardiac output, bone health parameters like bone mineral content, and biochemical markers including total cholesterol and glucose tolerance. Heart rate in both maximal and resting states, along with inflammatory markers like leptin, constituted integral components of the health-related outcomes.

### Study risk of bias assessment

To evaluate the risk of bias in the encompassed studies, this systematic review employed the Physiotherapy Evidence Database (PEDro) scale—a validated and reliable tool for this purpose.^27,28^ Comprising 11 items, with 10 contributing to each article's overall score, the scale assigns scores ranging from 0 (indicating the lowest quality) to 10 (indicating the highest quality).

Articles were qualitatively categorized based on their PEDro scale scores, following established cut-off points, into the following classifications: “poor” (<4 points), “fair” (4–5 points), “good” (6–8 points), and “excellent” (9–10 points).

For consistency and accuracy, two authors (TL and QX) independently reviewed and rated each included article according to the PEDro scale. Subsequently, these authors shared their scores and engaged in thorough discussions to address any discrepancies point by point. In cases where a consensus could not be reached, a third author (FMC) was consulted to provide their score and make the final decision, ensuring the resolution of any disagreements and fortifying the robustness of the risk of bias assessment.

## Results

### Study identification and selection

**Figure 1.**
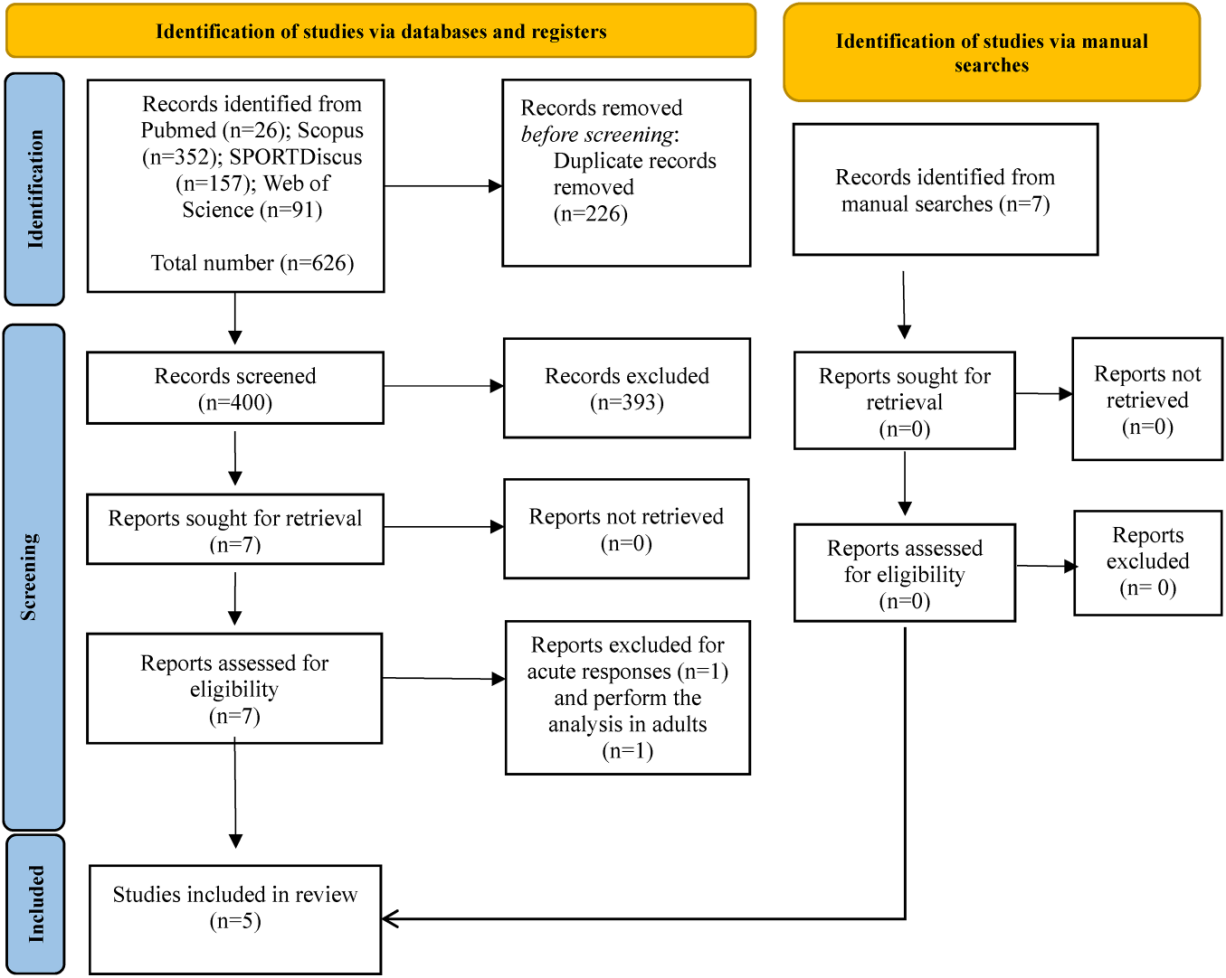
PRISMA flow diagram.

The initial search process identified a total of 626 titles (Figure 1). After eliminating duplicates (226 titles) through both automated and manual processes, 400 unique titles remained for further screening. These titles underwent evaluation for relevance based on their titles and abstracts, leading to the exclusion of 393 papers. The full texts of the remaining seven titles were then examined to determine their eligibility according to the predefined criteria.

PRISMA: Preferred Reporting Items for Systematic Reviews and Meta-Analyses.

Studies by Román et al.^29^ and Gomes et al.^30^ were excluded due to their focus on acute responses and the inclusion of adult professionals, respectively. Following the full-text screening process, a total of five articles were ultimately included in the systematic review.

### Risk of bias in studies

Among the five studies included in the systematic review, three studies received a quality score ranging from 4 to 5 points, indicating a moderate level of quality. On the other hand, the remaining two studies scored between 6 to 8 points, denoting a high level of quality (see Table 3). It is important to note that all articles shared a common limitation, as none of them reported information regarding blinding procedures for subjects, researchers, and assessors.

**Table 3.** Physiotherapy evidence database (PEDro) scale ratings.

	C1	C2	C3	C4	C5	C6	C7	C8	C9	C10	C11	Score
Arslan et al.^12^	1	1	0	0	0	0	0	0	1	1	1	4
Delextrat et al.^20^	1	1	1	1	0	0	0	1	0	1	1	6
Delextrat et al.^21^	1	1	1	1	0	0	0	0	0	1	1	5
Hassan et al.^31^	1	1	1	0	0	0	0	1	1	1	1	6
Zeng et al.^13^	1	1	1	0	0	0	0	1	0	1	1	5

C1: eligibility criteria were specified; C2: subjects were randomly allocated to groups; C3: allocation was concealed; C4: the groups were similar at baseline regarding the most important prognostic indicators; C5: there was blinding of all subjects; C6: there was blinding of all therapists who administered the therapy; C7: there was blinding of all assessors who measured at least one key outcome; C8: measures of at least one key outcome were obtained from more than 85% of the subjects initially allocated to groups; C9: all subjects for whom outcome measures were available received the treatment or control condition as allocated, or, where this was not the case, data for at least one key outcome were analyzed according to “intention to treat”; C10: the results of between-group statistical comparisons are reported for at least one key outcome; C11: the study provides both point measures and measures of variability for at least one key outcome.

### Study characteristics

Table 4 provides an overview of the current study's characteristics. All experiments were randomized parallel study designs. The parallel study design included a total of 10 individual groups, consisting of five SSG-based groups, four HIIT-based groups, and one control group. This review involved a total of 113 participants, with 56 participants in the SSG-based groups, 45 participants in the HIIT-based groups, and 12 participants in the passive control group. Four of the included studies focused on adolescent male basketball players, while one study focused on adolescent female^13^ basketball players.

**Table 4.** Characteristics of the included studies and outcomes extracted.

Study	*N*	Age (years old)	Sex	Experimental groups (*n*)	Parallel groups (*n*)	Randomization	Study duration (w)	Outcomes extracted	Instruments/tests for measuring the outcomes	Funding sources/project	Country where each study was conducted
Arslan et al.^12^	32	14.5 ± 0.5	Male	SSGs (*n* = 16)	HIIT (*n* = 16)	Yes	6	VIFT (km/h); distance covered at YYIRT-L1 (m); 5-, 10-, 20-, 30-m sprint time (s); CMJ height (cm); SJ height (cm); DJ height (cm); RSA total time (s); COD time (s)	30-15IFT; YYIRT-L1; 30-m linear sprint test; SJ; CMJ; DJ; RSA (6 × 2 × 15 m/20 s rest); T-drill COD test	Project UIDB/50008/2020.	Turkey
Delextrat et al.^20^	18	16.0–16.3	Male	SSGs (*n* = 9)	HIIT (*n* = 9)	Yes	6	VIFT (km/h); RSAtotal (s); RSA ideal time (s); RSA decrement (%); upper body power (m)	30-15IFT; RSA (6 × 2 × 10 m/20 s rest); 3-kg medicinal ball 2-handled chest pass	Not reported	United Kingdom
Delextrat et al.^21^	20	14.3 ± 0.5	Male	SSGs (*n* = 10)	HIIT (*n* = 10)	Yes	6	VIFT (km/h); sprint distance at 15 s (m); decrement in repeated sprint sequence (%)	30-15IFT; repeated sprint sequences (2 × 15 s over 20-m/15 s rest)	Not reported	United Kingdom
Hassan et al.^31^	24	10.92 ± 0.79	Male	SSGs (*n* = 12)	Control (*n* = 12)	Yes	10	Rhythmization ability (n); orientation ability (n); ability to differentiate (cm); responsiveness ability (cm); ability to balance (*n*)	Kiko battery test	GRANT1,671	Kingdom of Saudi Arabia
Zeng et al.^13^	19	19.9 ± 1.1	Females	SSGs (*n* = 9)	HIIT (*n* = 10)	Yes	4	VIFT (km/h); RSA mean (s); RSA best (s); RSA decrement (%); 20-m sprint (s); CMJ height (cm); COD time (s)	30-15IFT; RSA (2 × 15 m/20 s rest); 20-m linear sprint; modified agility T-test	Grant No. 19YJC890050 and Grant No. LY18C110002	China

SSGs: small-sided games; HIIT: high-intensity interval training; VIFT: final velocity at 30–15 Intermittent Fitness test (30–15 IFT); YYIRT-L1: Yo-Yo Intermittent Fitness test level 1; SJ: squat jump test; DJ: drop jump test; CMJ: countermovement jump test; RSA: repeated sprint ability test; COD: change-of-direction.

Tables 5 and 6 present the specific details of the SSG-based interventions and the characteristics of the parallel and control groups, respectively. The duration of the interventions varied, with the shortest intervention lasting four weeks^13^ and the longest intervention lasting 10 weeks.^31^ The minimum number of intervention sessions was 12, and the maximum number of sessions was 40.^31^

**Table 5.** Characteristics of SSG-based programs in the included studies.

Study	Duration (w)	Frequency (d/w)	Total Sessions (*n*)	SSG formats	SSG pitch dimension (length × width)	SSG area per player (m^2^)	Sets (*n*)	Reps (*n*)	Work duration	Work intensity	Relief duration	Relief intensity
Arslan et al.^12^	6	3	18	2 vs. 2	NR	NR	2	2	2 min 30 s–4 min	>85% HRmax	2 min	Passive
Delextrat et al.^20^	6	2	12	2 vs. 2	NR	NR	2	2	3 min–4 min 15 s	NR	NR	NR
Delextrat et al.^21^	6	2	12	2 vs. 2	28 × 7.5-m	52.5 m^2^	2	2–3	3 min–4 min 15 s	NR	2 min	NR
Hassan et al.^31^	10	4	40	NR	NR	NR	4	4	20 min–30 min	NR	1 min	Passive
Zeng et al.^13^	4	3	12	2 vs. 2	15 × 14-m	52.5 m^2^	3	2–3	2 min 40 s–3 min 45 s	NR	2 min	Passive

SSGs: small-sided games; W: weeks; d/w: days per week; min, minutes; NR: not reported; Passive: passive recovery.

**Table 6.** Characteristics of the parallel groups.

Study	Duration (w)	Frequency (d/w)	Total sessions (*n*)	Work duration	Work intensity	Relief duration	Relief intensity	Sets (*n*)	Reps (*n*)	Recovery between sets (duration)	Recovery between sets (intensity)
Arslan et al.^12^	6	3	18	6 min–9 min	90–95% V_IFT_	15 s	Passive	2	NR	NR	NR
Delextrat et al.^20^	6	2	12	8 min–13 min	95% V_IFT_	15 s	Active	2	NR	NR	NR
Delextrat et al.^21^	6	2	12	8 min–13 min	95% V_IFT_	15 s	Active	2	NR	NR	NR
Hassan et al.^31^	10	4	40	NR	NR	NR	NR	NR	NR	NR	NR
Zeng et al.^13^	4	3	12	6 min–9 min	90–95% V_IFT_	15 s	Passive	3	NR	NR	NR

W: weeks;d /w/d: days per week; min: minutes; s: seconds; NR: not reported; VIFT: maximal velocity at 30–15 IFT: Passive: passive recovery; Active: active recovery.

### Results of the individual studies

Table 7 displays the outcomes of individual studies concerning the effects on linear sprint, change of direction performance, and repeated sprint ability. In studies investigating the impact of SSGs versus running-based HIIT on linear sprint, such as those by Arslan et al.^12^ and Zeng et al.,^13^ it was noted that both SSGs and HIIT led to significant within-group improvements in linear sprint in the study by Arslan et al.,^12^ although this improvement was not observed in the linear 20-m sprint, as seen in the case of Zeng et al.^13^ Additionally, in both studies (Arslan et al.^12^ and Zeng et al.^13^), no differences between groups were observed.

**Table 7.** Results of individual studies on repeated sprint ability, linear sprinting and change-of-direction performance.

Study	Variable	SSGs pre	SSGs post	%dif (post–pre)	Within-SSGs (*p*-value)	CG pre	CG post	%dif (post–pre)	Within-CG (*p*-value)	Between groups (*p*-value)	ES between groups (post)
Linear sprint											
Arslan et al.^12^	5 m sprint time (s)	1.16 ± 0.05	1.11 ± 0.04	−4.31%⇑	*p < .05	1.14 ± 0.07	1.09 ± 0.06	−4.39%⇑	*p < .05	*p* > .05	d = −0.318
Arslan et al.^12^	10 m sprint time (s)	1.96 ± 0.19	1.87 ± 0.18	−4.59%⇑	*p < .05	1.93 ± 0.13	1.82 ± 0.13	−5.70%⇑	*p < .05	*p* > .05	d = −0.301
Arslan et al.^12^	20 m sprint time (s)	3.47 ± 0.24	3.36 ± 0.22	−3.17%⇑	*p < .05	3.43 ± 0.21	3.30 ± 0.19	−3.79%⇑	*p < .05	*p* > .05	d = −0.306
Arslan et al.^12^	30 m sprint time (s)	5.41 ± 0.34	5.18 ± 0.34	−4.25%⇑	*p < .05	5.35 ± 0.17	4.86 ± 0.14	−9.16%⇑	*p < .05	*p* > .05	d = −1.601
Zeng et al.^13^	20 m sprint time (s)	3.77 ± 0.21	3.73 ± 0.18	−1.06%⇑	*p* > .05	3.74 ± 0.18	3.79 ± 0.1	1.34%⇓	*p* > .05	*p* > .05	d = 0.272
Change-of-direction											
Arslan et al.^12^	T-Drill (s)	12.56 ± 0.85	11.84 ± 0.73	−5.73%⇑	*p < .05	12.46 ± 0.62	12.26 ± 0.61	−1.61%⇑	*p < .05	*p* > .05	d = 0.598
Arslan et al.^12^	T-Drill_mod_ (s)	6.64 ± 0.39	6.50 ± 0.37	−2.11%⇑	*p < .05	6.65 ± 0.54	6.60 ± 0.55	−0.75%⇑	*p < .05	*p* > .05	d = 0.173
Zeng et al.^13^	COD time (s)	7.02 ± 0.36	6.55 ± 0.23	−6.69%⇑	*p < .05	7.00 ± 0.39	6.62 ± 0.18	−5.43%⇑	*p < .05	*p* > .05	d = 0.315
Repeated sprint											
Arslan et al.^12^	RSA_total_ (s)	36.87 ± 1.29	35.03 ± 1.30	−5.00%⇑	*p < .05	36.66 ± 0.83	34.65 ± 0.79	−5.47%⇑	*p < .05	*p < .05 favoring HIIT	d = −0.395
Delextrat et al.^20^	total time RSA (s)	27.9 ± 2.4	28.7 ± 1.9	2.87%⇓	*p* > .05	27.1 ± 1.9	27.0 ± 1.8	10.37%⇑	*p* > .05	*p* > .05	d = −0.889
Delextrat et al.^20^	ideal time RSA (s)	26.3 ± 1.9	26.9 ± 1.9	2.28%⇓	*p* > .05	26.1 ± 1.8	25.8 ± 1.7	−1.15%⇑	*p* > .05	*p* > .05	d = −0.648
Delextrat et al.^20^	RSA decrement (%)	5.83 ± 2.53	6.33 ± 4.26	8.59%⇓	*p* > .05	3.75 ± 1.99	4.35 ± 2.51	16.00%⇓	*p* > .05	*p* > .05	d = −0.373
Delextrat et al.^21^	sprint distance at 15 s (1^st^ trial) (m)	71.6 ± 3.8	74.2 ± 4.5	3.64%⇑	*p < .05	72.8 ± 5.9	74.7 ± 7.6	2.61%⇑	*p < .05	*p* > .05	d = 0.059
Delextrat et al.^21^	sprint distance at 15 s (2^nd^ trial) (m)	67.1 ± 5.8	71.9 ± 3.8	7.06%⇑	*p < .05	68.0 ± 5.5	72.0 ± 6.0	5.88%⇑	*p < .05	*p* > .05	d = 0.025
Delextrat et al.^21^	decrement in repeated sprint sequence (%)	7.4 ± 3.0	5.8 ± 1.6	−21.62%⇑	*p < .05	7.2 ± 4.8	2.7 ± 1.8	−62.50%⇑	*p < .05	*p* > .05	d = −1.754
Zeng et al.^13^	RSA_mean_ (s)	6.94 ± 0.24	6.79 ± 0.23	−2.16%⇑	*p < .05	7.02 ± 0.15	6.89 ± 0.12	−1.85%⇑	*p < .05	*p* > .05	d = 0.426
Zeng et al.^13^	RSA_best_ (s)	6.71 ± 0.21	6.58 ± 0.22	−1.94%⇑	*p < .05	6.79 ± 0.13	6.65 ± 0.13	−2.06%⇑	*p < .05	*p* > .05	d = 0.236
Zeng et al.^13^	RSA_dec_ (%)	3.32 ± 1.14	3.19 ± 1.06	−4.07%⇑	*p* > .05	3.39 ± 1.58	3.73 ± 1.24	8.90%⇓	*p* > .05	*p* > .05	d = 0.236

SSGs: small-sided games; CG: control group; #unexperienced group; RSA: repeated sprint ability; ES: calculation of Cohen's d for the postintervention comparison between SSGs and control groups.

Examining studies analyzing the impact of both SSGs and running-based HIIT on change-of-direction performance, as evidenced by Arslan et al.^12^ and Zeng et al.,^13^ within-group improvements were observed in both groups, with no differences noted between the groups.

Finally, regarding the impacts of both SSGs and running-based HIIT on repeated sprint tests, Arslan et al.^12^ and Zeng et al.^13^ reported significant within-group improvements in variables such as repeated sprint ability (RSA) total time and best time. In contrast, Delextrat et al.^20^ did not report within-group improvements in RSA variables. However, in the study by Delextrat et al.,^21^ improvements in the decrement in repeated sprint sequence (%) were significant in both groups, though not differing between the groups. It is noteworthy that in the case of Arslan et al.,^12^ the improvements in repeated sprint ability (total time) were significantly greater in running-based HIIT than in SSGs.

Table 8 presents the findings from individual studies focusing on the impact on aerobic performance, specifically the final velocity achieved in the 30–15 Intermittent Fitness Test. Across the studies, it is evident that improvements in the SSGs groups ranged from a minimum of 4.07%, as observed in the studies by Delextrat et al.^20^ and Delextrat et al.,^21^ to a maximum of 7.29%, as seen in the case of Arslan et al.^12^ In the instances of running-based HIIT, improvements varied from a minimum of 3.45%, as reported in the studies by Delextrat et al.^20^ and Delextrat et al.,^21^ to a maximum of 8.19% in Arslan et al.^12^

**Table 8.** Results of individual studies on aerobic performance.

Study	Variable	SSGs pre	SSGs post	%dif (post-pre)	Within-SSGs (*p*-value)	CG pre	CG post	%dif (post–pre)	Within-CG (*p*-value)	Between groups (*p*-value)	ES between groups (post)
Arslan et al.^12^	VIFT (km/h)	13.31 ± 0.63	14.28 ± 0.58	7.29%⇑	*p < .05	13.66 ± 0.77	14.78 ± 0.52	8.19%⇑	*p < .05	*p* > .05	d = 0.642
Delextrat et al.^20^	VIFT (km/h)	17.2 ± 1.7	17.9 ± 1.5	4.07%⇑	*p < .05	17.4 ± 0.7	18.0 ± 1.0	3.45%⇑	*p < .05	*p* > .05	d = 0.033
Delextrat et al.^21^	VIFT (km/h)	17.2 ± 1.7	17.9 ± 1.5	4.07%⇑	*p < .05	17.4 ± 0.7	18.0 ± 1.0	3.45%⇑	*p < .05	*p* > .05	d = 0.033
Zeng et al.^13^	VIFT (km/h)	16.2 ± 0.6	16.9 ± 0.5	4.32%⇑	*p < .05	16.1 ± 0.6	16.8 ± 0.6	4.35%⇑	*p < .05	*p* > .05	d = −0.126

SSGs: small-sided games; CG: control group; #unexperienced group; VIFT: final velocity at 30–15 Intermittent Fitness Test; ES: calculation of Cohen's d for the post-intervention comparison between SSGs and control groups.

It is noteworthy that in the studies conducted by Arslan et al.,^12^ Delextrat et al.,^20^ Delextrat et al.,^21^ and Zeng et al.,^13^ both SSGs and running-based HIIT demonstrated significant within-group improvements, with no significant differences found between the groups.

Table 9 displays the data extracted from Hassan et al.^31^ regarding harmonic abilities. The findings indicate that both the SSGs group and the control group (not exposed to a specific intervention) demonstrated improvements in harmonic abilities over time. However, notable differences between the groups emerged, revealing significant improvements in favor of those participating in the SSGs group.

**Table 9.** Results of individual studies on harmonic abilities.

Study	Variable	SSGs pre	SSGs post	%dif (post–pre)	Within-SSGs (*p*-value)	CG pre	CG post	%dif (post–pre)	Within-CG (*p*-value)	Between groups (*p*-value)	ES between groups (post)
Hassan et al.^31^	Rhythmization ability (*n*)	10.17 ± 0.72	5.50 ± 0.52	−46.13%⇑	*p < .05	10.17 ± 0.83	7.00 ± 0.60	−31.21%⇑	*p < .05	*p < .05 favoring SSGs	d = 2.380
Hassan et al.^31^	Orientation ability (*n*)	3.42 ± 0.51	7.25 ± 0.87	112.87%⇑	*p < .05	3.33 ± 0.49	5.17 ± 0.39	55.26%⇑	*p < .05	*p < .05 favoring SSGs	d = −2.609
Hassan et al.^31^	Ability to differentiate (cm)	18.96 ± 0.20	10.50 ± 0.37	−44.42%⇑	*p < .05	18.92 ± 0.20	15.78 ± 0.58	−16.57%⇑	*p < .05	*p < .05 favoring SSGs	d = 8.857
Hassan et al.^31^	Responsiveness ability (cm)	179.50 ± 3.87	122.58 ± 2.39	−31.71%⇑	*p < .05	180.75 ± 4.02	163.42 ± 7.40	−9.59%⇑	*p < .05	*p < .05 favoring SSGs	d = 7.067
Hassan et al.^31^	Ability to balance (*n*)	9.33 ± 0.49	16.42 ± 0.67	75.61%⇑	*p < .05	9.25 ± 0.45	13.25 ± 0.45	43.24%⇑	*p < .05	*p < .05 favoring SSGs	d = −3.436

SSGs: small-sided games; CG: control group; #unexperienced group; ES: calculation of Cohen's d for the post-intervention comparison between SSGs and control groups.

## Discussion

### Overall findings

This systematic review, investigating SSG-based programs implemented on youth basketball players, uncovered consistent findings across studies indicating their effectiveness in improving aerobic performance and change of direction. However, contradictory findings surfaced concerning the impact on repeated sprint ability and linear sprint. Moreover, when compared to running-based HIIT, the studies indicated a tendency for SSGs to yield similar effects across various physical performance outcomes explored.

SSGs are typically played in compact formats and on small-to-medium-sized courts, often leading to highly intense cardiorespiratory efforts. These efforts result in varied heart rate responses, ranging from approximately 83% to 90.5% of maximal heart rate, depending on the format used and other task conditions.^24,32,33^ Given the intensity of this stimulus, it is anticipated that the cardiorespiratory system will undergo significant stress due to metabolic costs,^34^ thereby fostering adaptations.

For instance, in the study by Delextrat et al.,^21^ mean heart rate values of 90.5 ± 2.2% of HRpeak and 90.6 ± 2.2% of HRpeak were recorded for HIIT and SSG, respectively. Considering that prolonged exposure to the most demanding heart rate zones has been consistently linked to significant improvements in aerobic capacity,^35,36^ it is robust to suggest that SSGs can be as effective as running-based HIIT in enhancing aerobic performance with a minimum intervention period of four weeks as in the case of Zeng et al.^13^ up to six weeks as in the cases of Arslan et al.^12^ Delextrat et al.,^20^ and Delextrat et al.^21^

Another consistently observed result pertained to the effectiveness of SSG training programs in enhancing change of direction. Given that these games are typically conducted in confined spaces with fewer players, the heightened engagement required in the game leads to an increased number of technical actions.^37^ Consequently, individual behavior for creating spaces and opportunities^38^ contributes to a higher frequency of accelerations and decelerations.^33^ Given that these actions frequently unfold within the dynamics of gameplay, it is plausible that SSGs contribute to the development of the ability to recognize stimuli and enhance the capacity to decelerate, break if necessary, and swiftly reaccelerate in a different direction.^39^ This skill is crucial for avoiding opponents, creating better opportunities for receiving, or intercepting opponents.

In the studies conducted by Arslan et al.^12^ and Zeng et al.,^13^ which reported change-of-direction measures, both affirmed that SSGs proved to be as effective as running-based HIIT in enhancing change-of-direction performance, with improvements ranging between 5% and 6%. However, it is important to acknowledge that in other sports,^40^ variations in the number of turns and accelerations/decelerations during SSGs have been reported which may affect the individualization and control of the stimulus. Therefore, when prescribing SSGs to target this ability, it is advisable to progress along a continuum using both analytical scenarios and more ecological ones.^39^

Examining the impact of SSGs on repeated sprint ability revealed contradictory results. Arslan et al.^12^ and Zeng et al.^13^ reported significant within-group improvements, whereas the study by Delextrat et al.^20^ did not show such improvements. Moreover, in the study by Arslan et al.,^12^ running-based HIIT demonstrated significantly better improvements than SSGs. One challenge associated with SSGs is that smaller formats played in confined spaces may not enable players to reach their maximal velocity and sprint often.^24^ This limitation is noteworthy, especially in the context of repeated sprint ability. The early repetitions of repeated sprints demand improved sprint times, while the later repetitions require an enhanced aerobic metabolism.^41^ The contradictory findings in studies may be attributed to the absence of a necessary stimulus for improving sprinting. This insufficiency in enhancing linear sprint was further supported by the findings in the study conducted by Zeng et al.^13^ Hence, it is plausible that incorporating analytically focused repeated sprint training and sprint training may be necessary to complement the training and specifically the SSGs.^42^

### Implications for research

The present systematic review has unveiled a predominant focus on comparisons between SSG and other training methods, primarily centered around running-based HIIT. However, there is a notable absence of comparisons with alternative training modalities and a dearth of research exploring the integration of SSGs with other training approaches. Additionally, this systematic review emphasizes the imperative for further research, particularly in relation to considering the effects of the time of the season and trainability on the observed adaptations. Furthermore, there is a need for additional studies to strengthen the evidence base, especially in specific outcomes such as repeated sprint ability.

Moreover, future research in this area should focus on increasing the sample size, enhancing the diversity of the population (e.g. recruiting participants at different times and contexts), monitoring the load imposed by the intervention as well as the remaining sessions, and considering the comparison of various SSGs-based interventions (e.g. consistently comparing 2v2 vs. 4v4 across the entire program).

### Implications for practice

For practical applications, we recommend incorporating SSGs ranging from 2v2 to 4v4 to significantly contribute to the improvement of aerobic fitness over a minimum period of four weeks. Utilizing smaller formats and court sizes in SSGs is advisable to create more opportunities for frequent turns, accelerations, and decelerations. Conversely, larger formats played on expansive courts can be particularly beneficial for intensifying accelerations and achieving higher intensity levels, thus aiding in the enhancement of acceleration.

It is crucial for coaches not to restrict themselves solely to SSGs when aiming to enhance other vital physical performance measures such as repeated sprint ability or sprinting. Instead, coaches should employ the most appropriate and effective methods tailored to the specific requirements of these performance measures.

### Strengths and limitations

This systematic review is not without its limitations. One of the primary constraints is that, despite the growing number of studies, the pool of eligible studies remains relatively limited, which precludes the necessary consistency for conducting a meta-analysis. However, it is worth noting that, within the compiled studies, there was a consistent pattern in some variables, suggesting a similar trend in the evidence.

Another limitation stems from the inconsistency in reporting the training load of the sessions in the included studies. This lack of consistency hinders our ability to comprehend potential mechanisms explaining the observed adaptations. Additionally, most studies primarily employed a parallel design when compared to running-based HIIT, lacking a third control arm not exposed to any interventions. This absence makes it challenging to identify whether improvements are solely due to the interventions or influenced by external factors, given that players continue training in their primary in-field sessions.

## Conclusions

The present systematic review indicates that SSGs-based programs prove to be as effective as running-based HIIT in significantly improving aerobic performance with interventions lasting a minimum of four weeks. Similar effectiveness is observed in enhancing change of direction. Conversely, conflicting findings emerge regarding the effectiveness of SSGs-based programs in improving repeated sprint ability and linear sprint, although they generally yield results similar to those of running-based HIIT. Thus, basketball coaches can incorporate SSGs to promote a stimulus for tactical/technical behaviors, concurrently targeting aerobic stimulus to enhance overall capacity. However, it is advisable to explore alternative or complementary approaches specifically dedicated to improving repeated sprint ability or linear sprint.
